# Discovery of a novel lymphocytic choriomeningitis virus strain associated with severe human disease in immunocompetent patient, New Mexico

**DOI:** 10.1080/22221751.2025.2542250

**Published:** 2025-08-21

**Authors:** Jordan T. Gass, Robert A. Nofchissey, Frances M. Twohig, Chunyan Ye, Samuel M. Goodfellow, Kimiknu Mentore, Marcos Burgos, Oscar Negrete, Shannon Whitmer, John D. Klena, Deborah Cannon, Leanna Sayyad, Steven B. Bradfute

**Affiliations:** a University of New Mexico Health Sciences Center, Albuquerque, NM, USA; b Department of Systems Biology, Sandia National Laboratories, Livermore, CA, USA; c Centers for Disease Control and Prevention, Atlanta, GA, USA

**Keywords:** LCMV, lymphocytic choriomeningitis virus, *Mus musculus*, Sanger sequencing, phylogenetic analysis, New Mexico, mice, surveillance

## Abstract

Lymphocytic choriomeningitis virus (LCMV) is an underreported emerging pathogen and was recently included as a National Institute of Allergy and Infectious Disease (NIAID) priority pathogen. LCMV is an important cause of aseptic meningitis and has high morbidity and mortality during congenital and organ-transplant transmission. The house mouse, *Mus musculus*, is a reservoir for LCMV, and human infection occurs through exposure to aerosolized virus from the mouse excreta. In 2023, an immunocompetent patient in Albuquerque, New Mexico (NM), became infected with LCMV and experienced severe disease, representing a unique disease presentation as LCMV rarely causes disease in immunocompetent individuals. We trapped 20 *Mus musculus* in the patient’s home and tested for LCMV prevalence using PCR and ELISA. We found that 17/20 mice had LCMV RNA in their livers, while 3/20 had detectable anti-LCMV antibodies. We sequenced the complete coding region of the LCMV genome, revealing a novel strain, LCMV-ABQ. This represents the first documented LCMV strain from New Mexico. The S segment nucleotide sequence of LCMV-ABQ was 87.28% homologous to the closest related LCMV (strain 201102714), and the L segment was 82.03% homologous to strain Traub. Phylogenetic analysis revealed that LCMV-ABQ falls into lineage I. We surveyed 69 additional *Mus musculus* across four distinct sites in New Mexico, none of which were PCR-positive for LCMV. Thus, the patient’s home represents a hot spot for LCMV prevalence in New Mexico. This work informs the distribution and genetics of LCMV, which is increasingly recognized as an emerging pathogen of public health concern.

## Introduction

*Mammarenavirus choriomeningitidis*, more commonly known as lymphocytic choriomeningitis virus (LCMV), is the prototypical member of the *Arenaviridae* family and has recently been added as a NIAID priority pathogen [1, 2]. Arenaviruses are divided into Old World or New World viruses based on antigenicity, phylogenetic analysis, and geographic distribution [3]. LCMV belongs to the Old-World Arenaviruses [4] and is an enveloped virus with an ambisense RNA genome containing two segments, S (3.4 kb) and L (7.2 kb) [5]. Both segments employ an ambisense coding strategy to synthesize two proteins in opposite orientations, separated by a non-translated intergenic region (IGR) [6, 7]. The S segment encodes for the viral nucleoprotein (NP) and the glycoprotein precursor (GPC), which is post-translationally cleaved to generate three subunits, GP1, GP2, and transmembrane stable signal peptide, SSP [8, 9]. The L segment encodes for the viral RNA-dependent RNA polymerase (RdRp, or L protein) and a small zinc-binding matrix protein (Z) [10].

LCMV is a zoonotic virus which is maintained in rodents. The house mouse, *Mus musculus*, is thought to be the primary reservoir for LCMV, but LCMV antibodies have been found in a variety of different rodents, including hamsters, wood mice, rats, voles, squirrels, and shrews [11, 12]. LCMV is found globally due to the wide distribution of *Mus musculus*, which harbor chronic, asymptomatic LCMV infections and shed large quantities of the virus in their secreta and excreta [13]. Human LCMV infections can be congenital or acquired through inhaling aerosolized virus or contact with contaminated fomites [14]. If LCMV is congenitally acquired, there is a substantial risk of spontaneous abortion early in the pregnancy, and children born with LCMV suffer nervous system impairment [15]. Acquired LCMV infection is an important cause of aseptic meningitis and severe LCMV infections have been reported in immunocompromised individuals receiving organ transplants [16-18]. However, most acquired LCMV infections in immunocompetent persons result in mild, self-limited, or asymptomatic illness [19]. As a result, LCMV exposures and infections are underreported and underdiagnosed [20]. In March 2023, a 35-year-old woman in Albuquerque, New Mexico (NM), USA, was hospitalized and diagnosed with LCMV. The patient showed no signs of being immunocompromised, yet developed severe disease, representing an unusual disease presentation for LCMV. Her diagnosis came after anti-LCMV IgM and IgG antibodies were detected in her serum, and it was subsequently discovered that her housing had a significant rodent infestation.

In this study, we investigated the source of the patient’s LCMV exposure by trapping 20 *Mus musculus* in her residence 2 months after she was hospitalized. We characterized the entire coding region of the genome of a virus from one of the sampled rodents and compared the relatedness of this sequence to published LCMV sequences. We also compared LCMV prevalence in mice at the residence to mice collected from multiple sampling sites across NM.

## Methods

### Ethics statement

Collection procedures were performed according to the animal care and use guidelines of the American Society of Mammalogists [21] and approved by the University of New Mexico Health Sciences Center Institutional Animal Care and Use Committee. Additionally, rodent specimens were collected according to best practices for emerging pathogen research [22]. Approval to use residual human specimens for public health surveillance purposes was approved by the CDC/NCEZID Human Subjects Team, and IRB approval was not required for this work.

### Rodent and tissue sample collection

Twenty *Mus musculus* (house mice) were trapped on-site over one night using 22 Sherman live traps (3” x 3.5” x 9” H. B. Sherman Co., Tallahassee, FL, USA) baited with a mixture of peanut butter and oats. Mice were euthanized with isoflurane and exsanguinated by cardiac puncture. The following tissues were collected: serum (from whole blood), brown fat, spleen, kidney, liver, heart, lung, salivary glands (submandibular and sublingual), muscle (gastrocnemius) colon (with feces), urinary bladder (if urine was present) and embryos from pregnant females. Mouse tissues were collected, snap-frozen, and stored at −80°C until processed for RNA isolation. Additional *Mus musculus* from four different sites in New Mexico were captured over the course of several years, from 2019 to 2023 (samples were stored at −80°C). These sites were chosen as part of a prior mouse surveillance study in the lab due to their distribution across NM. As a result of the opportunistic nature of this trapping, sampling effort was relatively varied (Supplemental Table 3). We captured a total of 105 *Mus musculus* from these sites and screened 69 of them for LCMV. Samples were screened for LCMV RNA in their livers using the pan-LCMV primers [23].

### ELISA

Each of the 20 mice captured at the patient’s home was tested for LCMV-NP IgG antibodies using an ELISA kit (Cat # AE-300200-1, Alpha Diagnostic International, San Antonio, TX, USA) according to the manufacturer’s instructions. Briefly, mouse serum was diluted 1:100 with working serum diluent, and 100 µl was added to pre-determined wells in the ELISA plate. The plate was incubated for 60 min, then washed four times before 100 µl of diluted anti-Mouse IgG HRP was added to each well. After a 30-min incubation, wells were washed five times, and 100 µl of TMB substrate was added to each well. After a 15-min incubation in the dark, 100 µl of stop solution was added to terminate the reaction. Absorbance was read using 450 nm using a Biotek ELX800 absorbance microplate reader (Express Lab Werks, Summerfield, FL, USA). A sensitivity control was included to indicate a threshold to distinguish between positive and negative readings.

### RNA extraction

RNA extraction of rodent liver tissue was performed using a QIAmp Viral RNA Mini Kit (Qiagen, Hilden, Germany) according to the manufacturer’s instructions, with slight modifications. Briefly, an average of 40 mg of frozen liver tissue was added to a bead beater tube preloaded with 1.0 g of 1.0-mm-diameter zirconia beads (catalog number 1107911zx; BioSpec, Bartlesville, OK, USA), 1.0 g of 2.0-mm-diameter zirconia beads (catalog number 11079124zx; BioSpec, Bartlesville, OK, USA), and 800 µl of AVL buffer. Tissue was bead beat using a Biospec Mini-Beadbeater-16 for 45 s, allowed to rest for 2 min and then bead beat for another 30 s. Homogenates were centrifuged for 7 min at 4000 RPM, pipetted into a 1.7 mL microcentrifuge vial, and centrifuged for 10 min at 7000 RPM to pellet and remove debris. The clear lysates were then pipetted into a fresh 1.7 mL microcentrifuge vial. Carrier RNA was added to the clear lysate, and RNA extraction proceeded according to the manufacturer’s instructions.

### cDNA synthesis and PCR LCMV screen

The initial screening for LCMV and the full genome sequencing were performed using liver tissues, while later PCR experiments were conducted to test multiple tissue types. Per manufacturer instructions, cDNA was produced using SuperScript II (Invitrogen, Waltham, MA, USA). Briefly, 5 µl of extracted RNA was combined with 1 µl of random (nonspecific) primers, 1 µl of dNTP, and 5 µl RT-qPCR grade H2O, followed by a 5-min incubation at 65°C. Tubes were placed on ice for 15 s, then combined with 4 µl of 5x First Strand Buffer, 2 µl of 0.1 M DTT, and 1 µl of RNAseOUT, incubated for 2 min, followed by 1 µl of the SuperScript II enzyme. The tubes were incubated at 65°C for 5 min, placed on ice for 15 s, then incubated at ambient temperature for 10 min. The cDNA was synthesized for 50 min at 42°C, and the reaction was terminated by incubating for 15 min at 70°C. To screen for LCMV, PCR was performed using pan-LCMV primers targeting a region of the nucleoprotein gene within the S segment (10 µM of LCMV1748F 5’ – AIATIATRCARTCCATRAGIGCRCA – 3’ and LCM2377R 5’ – TCIGGIGARGGITGGCCITAYAT – 3’ [23]) and standard reagents for Platinum® *Taq* DNA polymerase High Fidelity. The thermocycler parameters were as follows: a 94°C hold for 2 min, then 35 cycles of 94°C for 15 s, 55°C for 30 s, 72°C for 1 min, followed by a 4 °C hold. A 655-bp PCR product that corresponded to the nucleoprotein gene for LCMV was produced and Sanger sequenced (Azenta Life Sciences).

### *Mus musculus* species and subspecies determination

*Mus musculus* were identified based on morphology. A quarter of all mice sampled had their subspecies determined by amplifying the mitochondrial b gene via PCR. RNA and cDNA were isolated as described above. PCR was performed using 800 ng of cDNA, 10 µM of Forward 15334L, 5’ -CTTCATTTTTGGTTTACAAGAC-3' and Reverse L14724, 5’-TGATATGAAAAACCATCGTTG-3’ primers [24], and standard reagents for Platinum® Taq DNA polymerase High Fidelity. Thermocycler parameters were as follows: 94°C for 2 min, then 35 cycles: 94°C for 15 s, 55°C for 30 s, 68°C for 1 min, followed by a 4°C hold. PCR products were Sanger Sequenced (Azenta Life Sciences) to determine subspecies.

### Primer design, sanger sequencing, and genome assembly

A PCR-tiling approach was used to sequence LCMV-ABQ by designing primers to amplify overlapping regions of the genome (Supplemental Figure 1). Primers were designed by aligning LCMV strains from GenBank that were highly similar to the partial LCMV sequence obtained from the mice, generating a consensus sequence using Geneious Prime (version 2024.0.7), then using PRIMAL SCHEME to design primers based on the consensus sequence to generate amplicons of ∼600 bp. cDNA synthesis was done as described above, and PCR was performed using ∼200 ng/µl cDNA, 10 µM of each primer (Supplemental Table 1), and standard reagents for the GoTaq green master mix (catalog number M7123; Promega). Thermocycler conditions were identical to the initial screening with the addition of a 5-min extension at 72°C preceding the 4°C hold. DNA gel electrophoresis was done using 1% agarose gels with a 100 bp DNA ladder and imaged using a UVP Transilluminator (Analytik Jena, Upland, CA, USA). PCR products were purified using a PureLink™ Quick PCR Purification Kit (Invitrogen, Waltham, MA, USA) and Sanger sequenced (Azenta Life Sciences). The resulting sequences were clipped for quality and aligned in Geneious Prime against a reference genome (GenBank JN687949.1 for the S segment, DQ868488.1 for the L segment). Ultimately, 6 primer pairs for the S segment and 14 primer pairs for the L segment were required to amplify the full coding region of LCMV (Supplemental Table 1). Consensus sequences for the S and L segments were extracted from the aligned reads and submitted to GenBank (accession numbers PX021332-PX021333) using Geneious Prime.

### Phylogenetic analysis

The consensus sequences for the S and L segments from LCMV-ABQ were aligned with other full-length LCMV genomes from GenBank using the CLUSTAL Omega feature in Geneious Prime. The Bayesian Information Criterion in MEGA11 [25] was used to evaluate nucleotide substitution models. The best-fit model for both the S and L segments was a general time reversible model plus invariate sites plus gamma distribution with 4 rate categories. Phylogenetic analysis was performed using the Bayesian inference method using MrBayes version 3.2.1 in Geneious Prime [26]. Default parameters were used for the priors. Two independent runs were conducted with 10 million generations per run and trees sampled every 500 generations, and the first 25% of trees were discarded as burn-in. Bayesian posterior probabilities (PP) were used to assess node support. The complete genome of Lunk mammarenavirus identified from a *Mus minutoides* in Zambia in 2010 was used as an outgroup.

## Results

### Rodent sampling and LCMV detection

In March 2023, a 35-year-old woman in Albuquerque, NM, USA presented to the University of New Mexico Hospital with leg and back pain, headache, fever, generalized weakness, nausea, and vomiting. The patient had no history of immunodeficiency, and her white blood cell count and differential were within normal limits, indicating no evidence of immunosuppression or immunodeficiency. She tested negative for standard bacterial cultures and fungal (coccidiomycosis) infections. She was serologically tested for various viral infections, including HIV, HSV, EBV, HBV, HAV, HCV, and WNV, all of which were negative, except for HAV and WNV, for which the patient had IgG but no IgM antibodies. Given the lack of IgM antibodies, these agents were ruled out as causative agents in this acute infection. Additional testing for arboviruses was not done by clinicians because of the time of year the patient presented. Hantavirus infection was ruled out because the clinical manifestation did not match a cardiopulmonary syndrome. Because the patient presented with symptoms of aseptic meningitis and later revealed that she had a significant rodent infestation at her home, the patient’s blood was sent to the CDC to be tested for LCMV. The patient was seropositive for LCMV IgM and IgG antibodies (CDC, Atlanta, GA, USA). We attempted to sequence LCMV from the patient; however, we could not detect LCMV RNA in the patient’s single blood sample that was available. The patient’s symptoms resolved within 6-weeks after she was discharged from the hospital.

**Figure 1.**
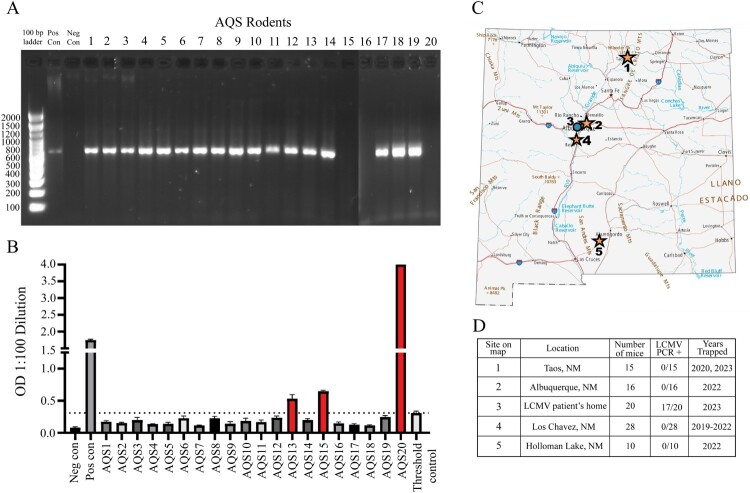
House mice (*Mus musculus*) captured at the LCMV patient’s home and across New Mexico were screened for LCMV. (A) Primers designed to amplify a 655 bp fragment of the LCMV NP were used to detect LCMV RNA [23]. Mice are denoted as AQS (Albuquerque South – location of patient’s home) 1-20. A negative rodent for LCMV was used as the negative control, and LCMV was used as the positive control. (B) An ELISA was used to screen for LCMV-specific IgG antibodies in *Mus musculus*. Optical densities higher than the threshold control were considered positive. (C) Map of New Mexico. *Mus musculus* were captured across New Mexico (yellow stars) and at the LCMV patient’s home (blue circle). Source: GIS Geography. (D) LCMV PCR results for the 89 total *Mus musculus* captured from five sites in New Mexico.

To investigate the source of the patient’s exposure to LCMV, we trapped mice at the patient’s residence in May 2023, achieving 91% trapping success (22 traps were set inside the home, and 20 mice were captured). We screened all 20 mice (AQS1-AQS20) for LCMV RNA in the liver lysates and anti-LCMV nucleoprotein antibodies in the serum. 17/20 mice were PCR positive for LCMV and 3/20 had anti-LCMV IgG nucleoprotein antibodies (Figure 1A,B). Two of the mice were antibody-positive and PCR-negative (AQS15 and AQS20), one mouse was antibody and PCR-positive (AQS13), and one mouse was antibody and PCR-negative (AQS16). Additional processing revealed that mice AQS11, AQS18, and AQS19 were LCMV positive in the kidney, heart, lung, salivary glands (AQS11), and brown fat (AQS18) (Supplemental Figure 2). One embryo was also tested from mouse AQS7 and was positive for LCMV RNA.

Given the high LCMV PCR positivity rate of mice captured at the patient’s home, we assessed 69 *Mus musculus* caught at four additional sites in New Mexico (Figure 1C, Supplemental Table 3). All 69 *Mus musculus* screened were PCR negative (Figure 1D, Supplemental Table 2, Supplemental Figure 3) in contrast to the 85% PCR positivity rate for the mice captured at the patient’s home in New Mexico. To determine whether LCMV positive mice were still present around the patient’s home, we set up multiple trap lines at variable distances from the home (Supplemental Table 4) two years after the initial trapping (June 2025). Despite multiple attempts, no *Mus musculus* were captured during this resampling.

### Sequencing the LCMV genome from captured *Mus musculus*

We obtained the complete LCMV coding region sequence (LCMV-ABQ) from liver lysates from one of the 20 *Mus musculus* (AQS11) captured at the home of the LCMV patient (accession numbers PX021332-PX021333). The amino acid length of each protein of LCMV-ABQ corresponds to previously published LCMV sequences. In addition to full viral genomes, our sequencing also detected non-complete viral genomes. Specifically, we detected a deletion viral genome that deleted the region between nucleotides 1559 and 1619, cutting off the end of the glycoprotein gene and the beginning of the IGR on the S segment. We amplified this region via PCR multiple times to gain a greater depth of coverage for this area, and many of these sequencing iterations confirmed the presence of the gap. LCMV deletion genomes, with deletions in this same region of the S segment, have been detected previously and are thought to play a role in viral interference to decrease viral replication [27].

### Phylogenetic analysis

**Figure 2.**
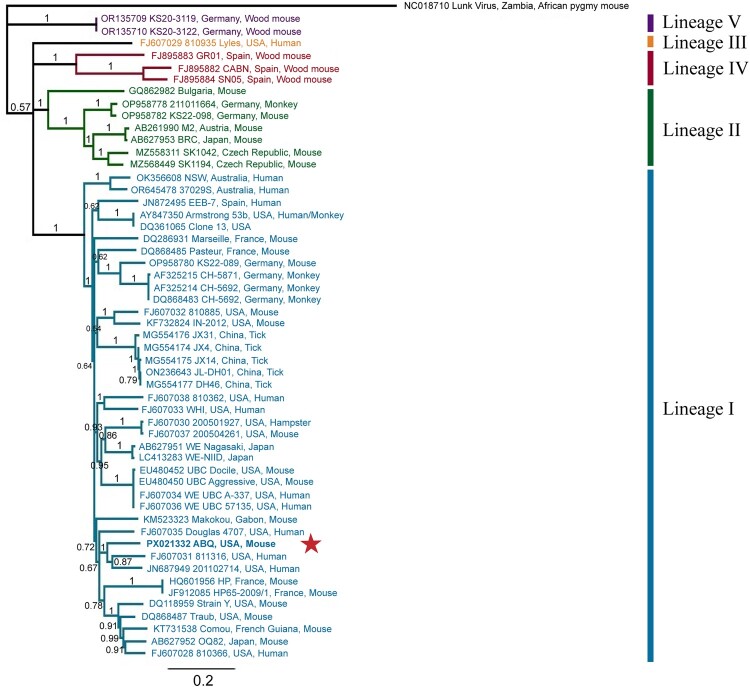
Phylogenetic analysis of the full-length S segment of LCMV sequences using Bayesian inference. This study's LCMV-ABQ S segment sequence was submitted to GenBank (accession number PX021332). LCMV-ABQ is bolded and designated with a red star. Bayesian posterior probabilities were used to assess node support. Lunk virus from *Mus minutoides* was used as an outgroup. Names of LCMV strains are composed of GenBank accession number, strain name, country of origin/isolation, and host species, if known.

**Figure 3.**
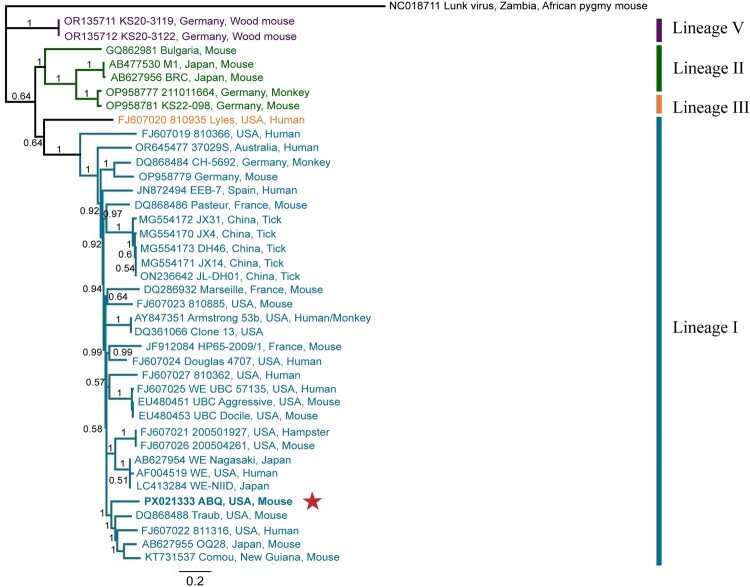
Phylogenetic analysis of the full-length L segment of LCMV sequences using Bayesian inference. This study's LCMV-ABQ L segment sequence was submitted to GenBank (accession number PX021333). LCMV-ABQ is bolded and designated with a red star. Bayesian posterior probabilities were used to assess node support. Lunk virus from *Mus minutoides* was used as an outgroup. Names of LCMV strains are composed of GenBank accession number, strain name, country of origin/isolation, and host species, if known.

We analysed the full coding regions of the S and L segments separately against other full-length LCMV genomes deposited in GenBank. LCMV-ABQ is bolded in each tree (Figures 2 and 3). The topology and branch support for each tree was further verified by doing Maximum Likelihood trees (Supplemental Figures 4 and 5). The S tree's topology is congruent with those generated in prior studies [28, 29], with strong support for the nodes on Lineages I, II, IV and V (P*P* = 1). Lineages I, II, and V on the L tree had highly supported positions (P*P* = 1) with lineage III having less support (*P* = 0.64). The LCMV-ABQ sequences cluster within lineage I on both trees, largely containing LCMV strains connected to *Mus musculus* and associated with human infections. The S segment nucleotide sequence similarity was highest between LCMV-ABQ and strain 201102714 (accession number JN687949), at 87.28% identity. The L segment nucleotide sequence similarity was highest between LCMV-ABQ and strain Traub (accession number DQ868488), at 82.03% identity. Fornuskova et al. have shown that the phylogenetic clades of LCMV are correlated with the geographic distribution and subspecies of *Mus musculus* [29]. A quarter of all mice captured had their mitochondrial cytochrome b gene sequenced, all of which were identified as *Mus musculus domesticus* (Supplemental Table 5).

Additionally, we compared the amino acid sequences of the four proteins (GP, NP, L, and Z) from LCMV-ABQ to other LCMV protein sequences in GenBank (Table 1). The L polymerase protein had the lowest amino acid sequence identity of the 4 proteins, having an amino acid sequence similarity of 89.59% to the closest LCMV strain, Traub (accession number DQ868488.1). The glycoprotein (GP) amino acid sequence similarity was the highest between LCMV-ABQ and strain Traub, having 97.36% identical residues. The nucleoprotein (NP) amino acid sequence similarity was equally high between strain Traub and strain 201102714, sharing 97.31% similar residues. The Z protein amino acid sequence similarity was the highest between LCMV-ABQ and strain Comou (accession number KT731537), sharing 92.22% similar residues.

**Table 1.** Amino acid sequence similarity of the LCMV-ABQ glycoprotein (GP), nucleoprotein (NP), polymerase (L), and small zinc-binding matrix protein (Z) compared to LCMV strains from each lineage. Gene sequences were aligned, and amino acid per cent identity matrices were generated using Geneious Prime (version 2024.0.7). Each protein's accession number and per cent identity are provided. Not published sequences denoted as “N.S.”

Accession Number			(%) Identity in amino acids with LCMV-ABQ			
Strain	(S:L)	Lineage	GP	NP	L	Z
Armstrong 53b	AY847350:AY847351	I	96.79	95.7	88.19	87.78
Comou	KT731538:KT731537	I	96.18	94.62	88	92.22
Marseille	DQ286931:DQ286932	I	96.18	95.16	87.19	77.78
OQ28	AB627952:AB627955	I	95.98	96.77	89.23	87.78
Traub	DQ868487:DQ868488	I	97.39	97.31	89.59	86.67
201102714	JN687949	I	96.79	97.31	N.S.	N.S.
811316	FJ607031:FJ607022	I	96.79	96.24	89.18	86.67
BRC	AB627953:AB627956	II	90.76	93.01	79.14	75.56
KS22-098	OP958782:OP958781	II	89.76	93.91	79.55	73.33
810935	FJ607029:FJ607020	III	89.76	91.58	79.1	79.12
CABN	FJ895882	IV	80.72	90.5	N.S.	N.S.
SN05	FJ895884	IV	79.92	90.5	N.S.	N.S.
KS20-3119	OR135709:OR135711	V	84.94	91.94	77.3	81.11
KS20-3122	OR135710:OR135712	V	84.94	91.94	77.34	81.11

## Discussion

LCMV is an important cause of aseptic meningitis, a teratogenic pathogen, and results in severe disease in transplant patients [12]. However, human cases are underreported since most infections result in asymptomatic or mild febrile illness, and even in instances of severe disease, many meningitis cases go undiagnosed and testing for LCMV is not considered [30]. Thus, determining the diversity of LCMV and its distribution in rodent hosts is of great public health interest. This study investigated LCMV infection of an immunocompetent patient who had close contact with house mice (*Mus musculus*) in Albuquerque, NM, in 2023. We isolated LCMV from *Mus musculus* captured at the patient’s home and sequenced the complete coding region of the genome, revealing an unreported LCMV strain, which we named LCMV-ABQ.

While almost all the mice captured at the patient’s home were positive for either LCMV RNA or anti-LCMV antibodies, sampling of *Mus musculus* in other parts of New Mexico revealed no additional LCMV-positive mice by PCR. However, it is possible that these mice may be antibody positive, as two of the mice captured at the patient’s home were antibody positive while being PCR negative, but we did not test for this. Nevertheless, most of the mice from the patient’s home were PCR positive, and PCR is a more sensitive test compared to serology, so the lack of PCR positive mice throughout the state suggests that the patient’s home represents a “hot spot” for LCMV prevalence. To our knowledge, this represents the first surveillance study for LCMV prevalence in *Mus musculus* from New Mexico. Additional studies are needed to further characterize the distribution of LCMV in wild mice in the Southwest United States.

Prior studies have shown a patchy distribution for LCMV-positive mice; Fornůsková et al. [29] sampled mice over 10 years across a 145 km by 110 km region stretching from northeastern Germany to the western Czech Republic and found that the small percentage of LCMV-positive mice were found in the same 12 km^2^ region. LCMV prevalence in wild *Mus musculus* varies geographically, with many studies showing a seroprevalence of <10% [29, 31-34]. Most mice in our study were RNA-positive and antibody-negative for LCMV, suggesting that using both PCR and serology provides a more accurate estimate of LCMV prevalence. Additionally, mouse density has been correlated with LCMV prevalence in urban surveillance studies [31, 32]. Our study supports this as the mice captured at the patient’s home had a high density (91% trap rate) and high prevalence of LCMV. This high prevalence among the mice at the patient’s home may result from the virus being maintained through vertical transmission and urban mice having limited dispersal distance [35].

From the 20 mice captured at the patient’s home, 17 were positive for LCMV RNA, and 3 were LCMV-antibody positive. Of the 3 mice that were antibody positive, 2 of them were not RNA positive, indicating that they likely were previously exposed to LCMV, developed an acute infection that generated antibodies, and have since cleared the virus. In contrast, mice with LCMV RNA but no antibodies likely have a chronic LCMV infection. These alternative immune responses in the reservoir host may reflect different viral transmission routes. When LCMV is vertically transmitted from mother to fetus, the mouse generates immune tolerance towards the virus, resulting in chronic, asymptomatic infection of adult mice, which shed large amounts of virus in their secretions [36]. Chronically infected mice can produce antibodies against the major structural proteins of LCMV [37]. However, these antibodies form complexes with viral antigens (present in excess in chronic infections), limiting the ability to detect antiviral antibodies via ELISA [38, 39]. In contrast, mice infected later in life through horizontal transmission generate antibodies against LCMV [35] and can clear the virus, largely through the action of CD8+ T cells [40]. Based on this, it is likely that most of the mice captured at the patient’s home were likely exposed to LCMV via vertical transmission, and thus produced chronic LCMV infections, while a few of the mice were only recently exposed to LCMV via horizontal transmission and were able to clear the virus and generate detectable antibodies. However, most of our knowledge on the murine immune response against LCMV has been gleaned from lab mice, with formal longitudinal studies on the immune response of wild mice infected with LCMV lacking to confirm these results.

We also compared the LCMV-ABQ genome to LCMV strains with well-characterized mutations linked to alterations in viral replication and infection outcomes. The LCMV strain Clone 13, and its parental strain, Armstrong 53b, differ by only 5 nucleotides and yet Armstrong results in acute infection in adult mice, whereas Clone 13 causes persistent infection [41]. These alternative disease outcomes are linked to 2 amino acid changes, one within the viral surface glycoprotein, GP1 (F260L), and one within the L polymerase (K1076Q) [42]. The GP1 subunit of the viral glycoprotein is responsible for binding to the host cell receptor, ɑ-dystroglycan (ɑ-DG), which is highly expressed on many cell types, especially on dendritic cells [43]. The change from phenylalanine to leucine promotes higher binding affinity, allowing the virus to displace other bound cellular components, such as laminin, promoting viral-receptor binding [44, 45]. This contributes to increased Clone 13 infection of dendritic cells, altering their function, thus limiting the host’s ability to generate a productive cytotoxic T lymphocyte (CTL) response and promoting viral persistence [43]. This GP1 F260L mutation is also present in other LCMV strains associated with persistent infection and diminished CTL responses, such as Traub, WE, and Pasteur [41]. Like Clone 13, LCMV-ABQ possesses a leucine at position 260 in the GP1, suggesting it likely has a high affinity for ɑ-DG. The other mutation associated with chronic infection in Clone 13 is the K1076Q mutation in the viral polymerase. This mutation enhances viral RNA replication, promoting higher viral loads relative to Armstrong [46, 47]. Despite sharing the GP1 F260L mutation with Clone 13, LCMV-ABQ shares the L polymerase mutation with Armstrong, suggesting it likely has lower replicative capacity compared to Clone 13.

In addition to the glycoprotein and the polymerase, amino acid changes mapped to the Z matrix protein and the nucleoprotein have been shown to alter viral replication and infection outcomes. It is well established that the LCMV NP counteracts the host type I IFN response, a function that is conserved among many arenaviruses [48]. Several residues have been linked to the anti-IFN function of NP, such as the DIEGR motif (D382, G385, R386) and the 3’-5” exonuclease motif (D382, E384, D459, H517, D522) [49-51]. LCMV-ABQ shares these conserved residues, suggesting that this strain can counteract the host type-I IFN response. Similarly, the Z matrix protein contains a conserved residue, W36, that contributes to the inhibitory activity of Z on RNA synthesis, and LCMV-ABQ contains a tryptophan at this residue [52].

Prior studies on the genetic diversity of LCMV have proposed that the LCMV S segment groups into five distinct lineages (I-V), while the L segment groups into four (I, II, III, V – the corresponding L segment of the lineage IV S segments was not sequenced) [28, 29, 53, 54]. Given the wide geographic range and historical movement of *Mus musculus*, these lineages do not group well based on geography or time of isolation [28, 29]. However, Fornůsková et al. have shown that the lineages group well according to host subspecies, with lineage I being associated with *M. musculus domesticus* (distributed across western and southern Europe, the Middle East, northern Africa, North America, South America, Australia, and Oceana) and lineage II with *M. musculus musculus* (found mostly in central and northern Europe) [29]. Both lineages IV and V are associated with the wood mouse, *Apodemus sylvaticus,* in Spain and Germany, respectively [29, 55]. Our phylogenies are highly congruent with previously published trees [28, 29, 53, 54, 56]. Both segments of the LCMV-ABQ genome fall within lineage I, which contains other LCMV strains linked with human infection. This work supports the hypothesis that LCMV lineages are grouped based on *M. musculus* subspecies, as LCMV-ABQ was isolated from a *M. musculus domesticus* (Supplemental Table 5) and groups within lineage I.

The LCMV-ABQ S segment had the highest per cent nucleotide similarity to LCMV strains 811316 and 201102714. Both strains 811316 and 201102714 are associated with clusters of human organ-transplant LCMV infections in the United States [18, 28]. Meanwhile, the LCMV-ABQ L segment had the highest per cent nucleotide similarity to LCMV Traub, a strain isolated from a mouse colony at the Rockefeller Institute for Medical Research in Princeton, New Jersey, in 1935 [57]. The next closest related strains were 811316 (discussed above), followed by OQ28 and Comou. Strain OQ28 was isolated in 1990 from a wild *Mus musculus* in Osaka, Japan [58], while strain Comou was isolated in 2013 during surveillance of *Mus musculus* in French Guiana [41, 53].

This study showcases the first published LCMV sequence from New Mexico. Surveys for LCMV infection in humans in New Mexico and surrounding states are lacking. However, some cases have been reported in the Southwestern U.S [20]. The presence of LCMV in New Mexico is further supported by a recent investigation of an immunocompromised individual diagnosed with LCMV after exposure to mouse droppings in his New Mexico residence [59]. LCMV is an important emerging pathogen, especially in regions with high exposure to house mice. Enhanced surveillance of rodents is essential to define the diversity, prevalence, and distribution of LCMV. Given the likelihood of significant numbers of undiagnosed LCMV infection in humans, there should be an emphasis on prospective and retrospective assessment of LCMV infection in aseptic meningitis.

## Supplementary Material

Supplemental Material

LCMV Bayes tree raw posterior output and summary stats.xlsx
