# Supplementary material for: Discovery of a novel lymphocytic choriomeningitis virus strain associated with severe human disease in immunocompetent patient, New Mexico
Source: Emerg Microbes Infect. 2025 Aug 21;14(1):2542250. doi: 10.1080/22221751.2025.2542250 (PMC12372502; doi:10.1080/22221751.2025.2542250)
Supplement: Supplemental Material [file TEMI_A_2542250_SM8126.docx]

**Supplementary Material**

**Tables**

**Supplemental Table 1**: PCR tiling primers used to Sanger-sequence lymphocytic choriomeningitis virus (LCMV) strain ABQ. Primers were generated using PRIMAL SCHEME software.

|  |  | Coverage | |  | Sense |
| --- | --- | --- | --- | --- | --- |
| Number | Primer sequence (5’ -> 3’) | Start | Stop | Name | strand |
| 1 | CCTAGGCTTTTTGGATTGCGCTT | 13 | 35 | LCMV_S_Segment_1_LEFT | + |
| 2 | ATTCCAAAAGGACCGGCATACC | 778 | 799 | LCMV_S_Segment_1_RIGHT | - |
| 3 | GAGCATAGTCTCCAGCTTACACCTC | 476 | 500 | LCMV_S_Segment_1.5_LEFT | + |
| 4 | CATTTGAGTTCCGCAGCAAGGATC | 929 | 952 | LCMV_S_Segment_1.5_RIGHT | - |
| 5 | TGGACAGGTTCAGATGGCAA | 687 | 707 | LCMV_S_Segment_2_LEFT | + |
| 6 | CCCTTTATATGCCTGTGTGTTGGTA | 1432 | 1456 | LCMV_S_Segment_2_RIGHT | - |
| 7 | CATAAAGAGACAAGGGAGCACTCC | 1334 | 1358 | LCMV_S_Segment_3_LEFT | + |
| 8 | CCCCCACATGGGTAGACATTG | 2148 | 2168 | LCMV_S_Segment_3_RIGHT | - |
| 9 | TGAGGCTTCCTTAGTCATCTCCAC | 1846 | 1870 | LCMV_S_Segment_4_LEFT | + |
| 10 | AATGCCAAGTTTGACGATGGCC | 2755 | 2776 | LCMV_S_Segment_4_RIGHT | - |
| 11 | TTGCCTCCGTCCAGTAAAGCTG | 2522 | 2543 | LCMV_S_Segment_5_LEFT | + |
| 12 | CGCACAGTGGATCCTAGGCATTT | 3355 | 3376 | LCMV_S_Segment_5_RIGHT | - |
| 13 | GGGATCCTAGGCGTTTAGTTGC | 8 | 30 | LCMV_L_Segment_1_LEFT | + |
| 14 | AGGTTCAGACTCAAGGGGAAGT | 610 | 631 | LCMV_L_Segment_1_RIGHT | - |
| 15 | CGGAGAGCCCGATCACTCGATC | 551 | 572 | LCMV_L_Segment_2_LEFT | + |
| 16 | GTGCCCTTGACTGTGAGAATGGGTG | 946 | 970 | LCMV_L_Segment_2_RIGHT | - |
| 17 | TGAATCGATGCAGGAGGAGGT | 808 | 829 | LCMV_L_Segment_3_LEFT | + |
| 18 | GGAGATGTTGTGTGCAATGCTG | 1438 | 1459 | LCMV_L_Segment_3_RIGHT | - |
| 19 | ACCTGGACTCTGTAATTGGCAC | 1238 | 1260 | LCMV_L_Segment_4_LEFT | + |
| 20 | AGTCCCAGGATTAAATTCCTTGACC | 1867 | 1891 | LCMV_L_Segment_4_RIGHT | - |
| 21 | GGACAGTATAATGCTCATCTCTCTTCC | 1780 | 1807 | LCMV_L_Segment_5/6_LEFT | + |
| 22 | GAAGCTATTCTCCAGCTGAGGG | 2632 | 2653 | LCMV_L_Segment_5/6_RIGHT | - |
| 23 | GGGAACATCTCATTCAAATTCAACCA | 2559 | 2584 | LCMV_L_Segment_7_LEFT | + |
| 24 | GATAGTGACCCAGAAGAAGTCCTTG | 3163 | 3187 | LCMV_L_Segment_7_RIGHT | - |
| 25 | TGTGTCTATTGTTTCACAAAGTTGATGT | 2957 | 2984 | LCMV_L_Segment_8_LEFT | + |
| 26 | GATGTGCCCATTTTTGTTTTTAATGCT | 3600 | 3626 | LCMV_L_Segment_8_RIGHT | - |
| 27 | TGTGCCAAGTTAACAAGGTGCT | 3520 | 3541 | LCMV_L_Segment_9_LEFT | + |
| 28 | TGGTCTGGATGAGATGGCAAAAA | 4173 | 4196 | LCMV_L_Segment_9_RIGHT | - |
| 29 | TCTTCTTCCAATTTGTCCCAGTCAA | 4020 | 4044 | LCMV_L_Segment_10_LEFT | + |
| 30 | TGCTTGTTAGTGCTGTGAGCC | 4687 | 4698 | LCMV_L_Segment_10_RIGHT | - |
| 31 | GGCACCAATGACCAACTTAGAGAC | 4577 | 4600 | LCMV_L_Segment_11_LEFT | + |
| 32 | CTATTTTTGGCACTGGTGAGAGGG | 5149 | 5172 | LCMV_L_Segment_11_RIGHT | - |
| 33 | GGTTGACAAAGAAACCAAACTCACT | 4999 | 4023 | LCMV_L_Segment_12_LEFT | + |
| 34 | AGTGGTGGAATGCAAAGAAGTGT | 5611 | 5633 | LCMV_L_Segment_12_RIGHT | - |
| 35 | GTAACACCTTGAAGATTCTCCAGTCT | 5534 | 5559 | LCMV_L_Segment_13_LEFT | + |
| 36 | GAATTTAGATGTCTTGTGTCTTTCTTCACT | 6141 | 6170 | LCMV_L_Segment_13_RIGHT | - |
| 37 | GTCCCACTAGTGATAAGCACCTTTGG | 5905 | 5930 | LCMV_L_Segment_14_LEFT | + |
| 38 | GGAAGTCTTAAGGGATGCAAGATCATCTC | 6556 | 6583 | LCMV_L_Segment_14_RIGHT | - |
| 39 | GGGTCTCATAAAGTTCAGAGAATTCCT | 6364 | 6390 | LCMV_L_Segment_15_LEFT | + |
| 40 | TTGATGCGCAATGGATGAGACT | 7181 | 7200 | LCMV_L_Segment_15_RIGHT | - |

**Supplemental Table 2**: *Mus musculus* captured at four additional sites in New Mexico were PCR negative for LCMV RNA using the same methods used to detect the LCMV positive mice at the patient’s home.

| Location | Sample ID | PCR Result | Date collected |
| --- | --- | --- | --- |
| Los Chavez, NM | LC-001 | Negative | 9/7/19 |
| Los Chavez, NM | LC-002 | Negative | 9/7/19 |
| Los Chavez, NM | LC-003 | Negative | 9/7/19 |
| Los Chavez, NM | LC-004 | Negative | 9/7/19 |
| Los Chavez, NM | LC-005 | Negative | 9/7/19 |
| Los Chavez, NM | LC-006 | Negative | 9/7/19 |
| Los Chavez, NM | LC-008 | Negative | 9/7/19 |
| Los Chavez, NM | LC-009 | Negative | 9/7/19 |
| Los Chavez, NM | LC-010 | Negative | 9/7/19 |
| Los Chavez, NM | LC-012 | Negative | 9/7/19 |
| Los Chavez, NM | LC-016 | Negative | 9/21/19 |
| Los Chavez, NM | LC-021 | Negative | 9/21/19 |
| Los Chavez, NM | LC-051 | Negative | 8/8/20 |
| Los Chavez, NM | LC-052 | Negative | 8/8/20 |
| Los Chavez, NM | LC-058 | Negative | 8/8/20 |
| Los Chavez, NM | LC-059 | Negative | 8/8/20 |
| Los Chavez, NM | LC-064 | Negative | 10/18/20 |
| Los Chavez, NM | LC-065 | Negative | 10/18/20 |
| Los Chavez, NM | LC-084 | Negative | 12/31/21 |
| Los Chavez, NM | LC-087 | Negative | 12/31/21 |
| Los Chavez, NM | LC-088 | Negative | 12/31/21 |
| Los Chavez, NM | LC-090 | Negative | 12/31/21 |
| Los Chavez, NM | LC-093 | Negative | 1/1/22 |
| Los Chavez, NM | LC-094 | Negative | 1/1/22 |
| Los Chavez, NM | LC-095 | Negative | 1/1/22 |
| Los Chavez, NM | LC-096 | Negative | 1/1/22 |
| Los Chavez, NM | LC-097 | Negative | 4/30/22 |
| Los Chavez, NM | LC-099 | Negative | 4/30/22 |
| Albuquerque, NM | ABQ-023 | Negative | 12/16/21 |
| Albuquerque, NM | ABQ-024 | Negative | 12/16/21 |
| Albuquerque, NM | ABQ-025 | Negative | 12/16/21 |
| Albuquerque, NM | ABQ-026 | Negative | 12/16/21 |
| Albuquerque, NM | ABQ-027 | Negative | 12/16/21 |
| Albuquerque, NM | ABQ-028 | Negative | 12/16/21 |
| Albuquerque, NM | ABQ-029 | Negative | 12/16/21 |
| Albuquerque, NM | ABQ-030 | Negative | 12/16/21 |
| Albuquerque, NM | ABQ-049 | Negative | 8/24/22 |
| Albuquerque, NM | ABQ-050 | Negative | 8/24/22 |
| Albuquerque, NM | ABQ-051 | Negative | 8/24/22 |
| Albuquerque, NM | ABQ-052 | Negative | 8/24/22 |
| Albuquerque, NM | ABQ-053 | Negative | 8/24/22 |
| Albuquerque, NM | ABQ-054 | Negative | 8/24/22 |
| Albuquerque, NM | ABQ-055 | Negative | 8/24/22 |
| Albuquerque, NM | ABQ-056 | Negative | 8/24/22 |
| Taos, NM | T-004 | Negative | 8/20/20 |
| Taos, NM | T-012 | Negative | 8/20/20 |
| Taos, NM | T-018 | Negative | 8/20/20 |
| Taos, NM | T-020 | Negative | 8/20/20 |
| Taos, NM | T-022 | Negative | 8/20/20 |
| Taos, NM | T-067 | Negative | 8/20/20 |
| Taos, NM | T-123 | Negative | 8/17/22 |
| Taos, NM | T-205 | Negative | 8/17/23 |
| Taos, NM | T-206 | Negative | 8/17/23 |
| Taos, NM | T-209 | Negative | 8/17/23 |
| Taos, NM | T-213 | Negative | 8/17/23 |
| Taos, NM | T-215 | Negative | 8/17/23 |
| Taos, NM | T-244 | Negative | 8/18/23 |
| Taos, NM | T-245 | Negative | 8/18/23 |
| Taos, NM | T-250 | Negative | 8/18/23 |
| Holloman Lake, NM | H-310881 | Negative | 3/3/22 |
| Holloman Lake, NM | H-310882 | Negative | 3/3/22 |
| Holloman Lake, NM | H-310883 | Negative | 3/3/22 |
| Holloman Lake, NM | H-310884 | Negative | 3/3/22 |
| Holloman Lake, NM | H-311864 | Negative | 3/3/22 |
| Holloman Lake, NM | H-311868 | Negative | 3/3/22 |
| Holloman Lake, NM | H-310958 | Negative | 5/5/22 |
| Holloman Lake, NM | H-310959 | Negative | 5/5/22 |
| Holloman Lake, NM | H-311909 | Negative | 5/5/22 |
| Holloman Lake, NM | H-311910 | Negative | 5/5/22 |

| Location | Date | Trap-Nights | Total *Mus musculus* trapped | Total *Mus musculus* tested for LCMV |
| --- | --- | --- | --- | --- |
| Los Chavez | 09/07/2019 | 26 | 11 | 10 |
| Los Chavez | 09/21/2019 | 16 | 6 | 2 |
| Los Chavez | 8/8-8/11/2020 | 56 | 7 | 4 |
| Los Chavez | 10/18/2020 | 15 | 3 | 2 |
| Los Chavez | 12/31/2021-1/1/2022 | 32 | 13 | 8 |
| Los Chavez | 4/30/2022 | 14 | 6 | 2 |
| **Total Los Chavez** | **2019-2023** | **159** | **46** | **28** |
| Albuquerque | 12/16/21 | 40 | 8 | 8 |
| Albuquerque | 08/24/22 | 40 | 8 | 8 |
| **Total Albuquerque** | **2021-2022** | **80** | **16** | **16** |
| Taos | 8/20/2020 – 8/21/2020 | 80 | 9 | 6 |
| Taos | 8/17/2022 | 80 | 6 | 1 |
| Taos | 8/17/2023-8/18/2023 | 80 | 8 | 8 |
| **Total Taos** | **2020-2023** | **240** | **23** | **15** |
| Holloman Lake | 3/3/2022 | 70 | 6 | 6 |
| Holloman Lake | 5/5/2022 | 386 | 14 | 4 |
| **Total Holloman Lake** | **2022** | **456** | **20** | **10** |
|  |  |  | **105** | **69** |

**Supplemental Table 3:** Trapping effort for the additional *Mus musculus* trapped in New Mexico (NM). Traps-Nights represents the total traps set multiplied by the number of nights that traps were set out. A total of 105 mice were trapped across 4 sites in NM and 69 of these were tested for LCMV using PCR.

**Supplemental Table 4:** Additional mouse traps were set at various distances from the patient’s home in June 2025 to resample *Mus musculus* from the same area where the LCMV positive mice were detected in May 2023. Traps-Nights represents the total traps set multiplied by the number of nights that traps were set out.

| **Additional trapping location** | **Trap-Nights** | **Number of *Mus musculus* trapped** | **Distance from LCMV patient’s house** |
| --- | --- | --- | --- |
| Albuquerque site 1 | 30 | 0 | <1 mile |
| Albuquerque site 2 | 28 | 0 | 4.4 miles |
| Albuquerque site 3 | 88 | 0 | 7.3 miles |
| Albuquerque site 4 | 24 | 0 | 9.8 miles |

**Supplemental Table 5:** *Mus musculus* subspecies were confirmed for at least 25% of the mice captured at each site by sequencing the mitochondrial cytochrome b gene**.**  Cytochrome b sequences were obtained for selected mice trapped in different locations. Table shows the top hit of these sequences when analyzed by NCBI blastn.

| Location | Sample ID | Species |
| --- | --- | --- |
| Los Chavez, NM | LC-001 | *Mus musculus domesticus* |
| Los Chavez, NM | LC-002 | *Mus musculus domesticus* |
| Los Chavez, NM | LC-003 | *Mus musculus domesticus* |
| Los Chavez, NM | LC-004 | *Mus musculus domesticus* |
| Los Chavez, NM | LC-005 | *Mus musculus domesticus* |
| Los Chavez, NM | LC-006 | *Mus musculus domesticus* |
| Los Chavez, NM | LC-009 | *Mus musculus domesticus* |
| Los Chavez, NM | LC-012 | *Mus musculus domesticus* |
| Albuquerque, NM | ABQ-023 | *Mus musculus domesticus* |
| Albuquerque, NM | ABQ-024 | *Mus musculus domesticus* |
| Albuquerque, NM | ABQ-027 | *Mus musculus domesticus* |
| Albuquerque, NM | ABQ-029 | *Mus musculus domesticus* |
| Albuquerque, NM | ABQ-030 | *Mus musculus domesticus* |
| Albuquerque, NM | ABQ-049 | *Mus musculus domesticus* |
| Albuquerque, NM | ABQ-050 | *Mus musculus domesticus* |
| Albuquerque, NM | ABQ-056 | *Mus musculus domesticus* |
| Taos, NM | T-004 | *Mus musculus domesticus* |
| Taos, NM | T-018 | *Mus musculus domesticus* |
| Taos, NM | T-123 | *Mus musculus domesticus* |
| Taos, NM | T-244 | *Mus musculus domesticus* |
| Taos, NM | T-245 | *Mus musculus domesticus* |
| Holloman Lake, NM | H-310882 | *Mus musculus domesticus* |
| Holloman Lake, NM | H-310883 | *Mus musculus domesticus* |
| Holloman Lake, NM | H-310884 | *Mus musculus domesticus* |
| Holloman Lake, NM | H-311868 | *Mus musculus domesticus* |
| Holloman Lake, NM | H-311909 | *Mus musculus domesticus* |
| LCMV patient’s home, NM | AQS5 | *Mus musculus domesticus* |
| LCMV patient’s home, NM | AQS7 | *Mus musculus domesticus* |
| LCMV patient’s home, NM | AQS11 | *Mus musculus domesticus* |
| LCMV patient’s home, NM | AQS15 | *Mus musculus domesticus* |
| LCMV patient’s home, NM | AQS18 | *Mus musculus domesticus* |
| LCMV patient’s home, NM | AQS19 | *Mus musculus domesticus* |
| LCMV patient’s home, NM | AQS20 | *Mus musculus domesticus* |

**Supplementary Figures**

**Supplemental Figure 1**: Confirmation of product for LCMV amplification using PCR tiling primers. A) Schematic of sequencing strategy to capture the LCMV genome. Horizontal lines represent obtained PCR products. Primer names are indicated on each line and correspond with Supplemental Table 1. B) Gels displayed show bands for each primer pair. These bands were purified, and Sanger sequenced to confirm the LCMV-ABQ genome. A 100 bp ladder (left) was used to demonstrate expected band length.

**Supplemental Figure 2**: Primers designed to amplify a 655 bp fragment of the LCMV NP [20] were used to detect LCMV RNA in multiple tissue types from select mice captured at the patient’s home. Tissues are color-coded by mouse (AQS11-blue, AQS18-orange, AQS19-purple, AQS7-green). A 100 bp ladder (left) was used to demonstrate the expected band length.


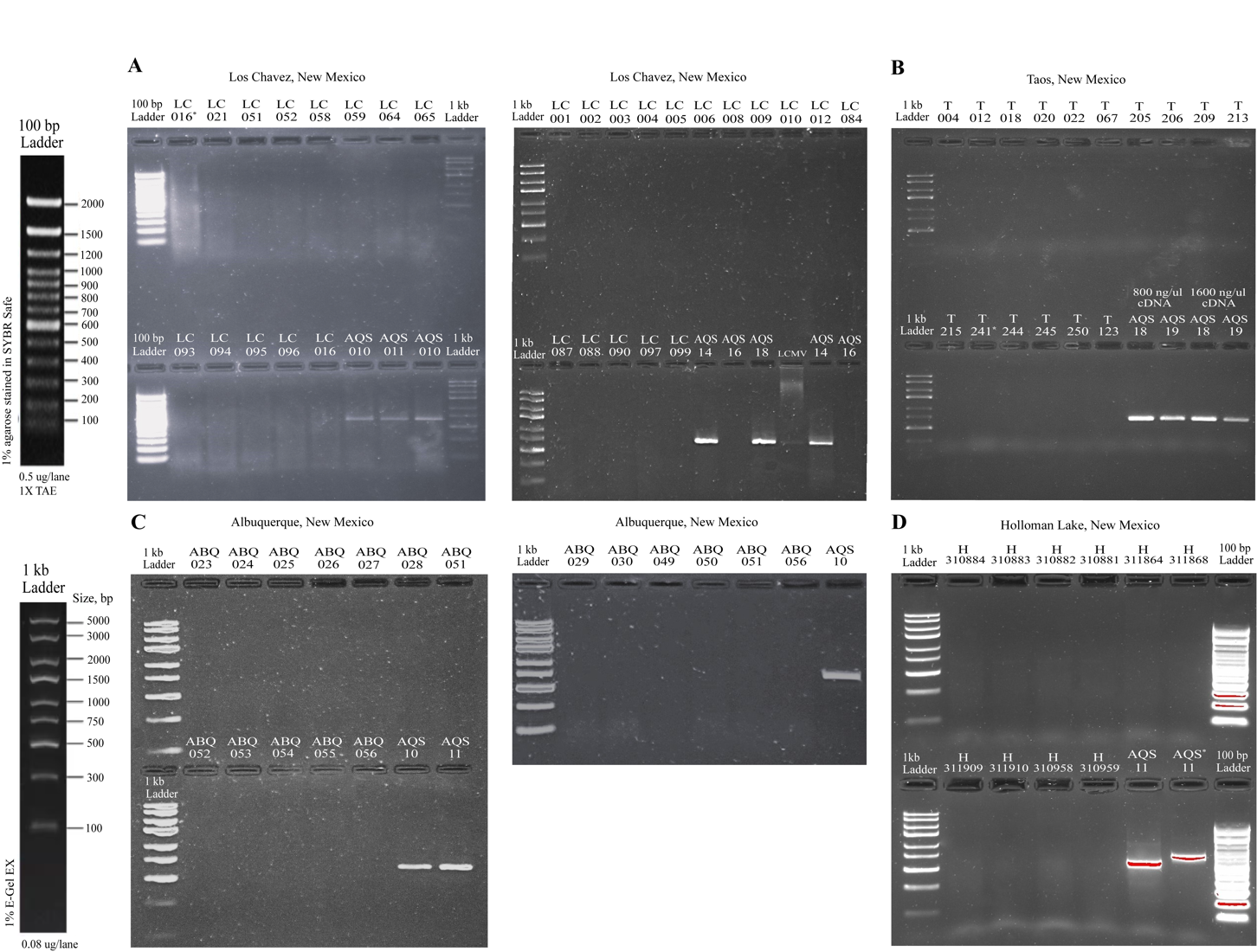


**Supplemental Figure 3:** Confirmation of negative LCMV *Mus musculus* using pan-LCMV primers designed to amplify a 655 bp fragment of the LCMV NP RNA [20]. A 100 bp ladder (top left) or 1 kb ladder (bottom left) was used to demonstrate band length. A) Mice collected from Los Chavez, NM, are designated ‘LC’. Positive control mice from the LCMV patient’s home are AQS-10, -11, -14, and -18. Mouse AQS16 is a negative control. ‘LCMV’ is strain Armstrong. PCR for LC016* was done with 10x the concentration of cDNA. B) Mice collected from Taos, NM, are designated ‘T’. Positive controls are AQS18 and AQS19 (PCR done using either 800 ng/µl or 1600 ng/µl cDNA). T241* is from a *Peromyscus sonoriensis*. C) Mice collected from Albuquerque, NM (not at the patient’s home) are designated ‘ABQ’. Positive controls are AQS10 and AQS11. D) Mice collected from Holloman Lake, NM are designated ‘H’. AQS11 and AQS11* are positive controls. AQS11* was done using the S segment 3 primers (Supplemental Table 1).


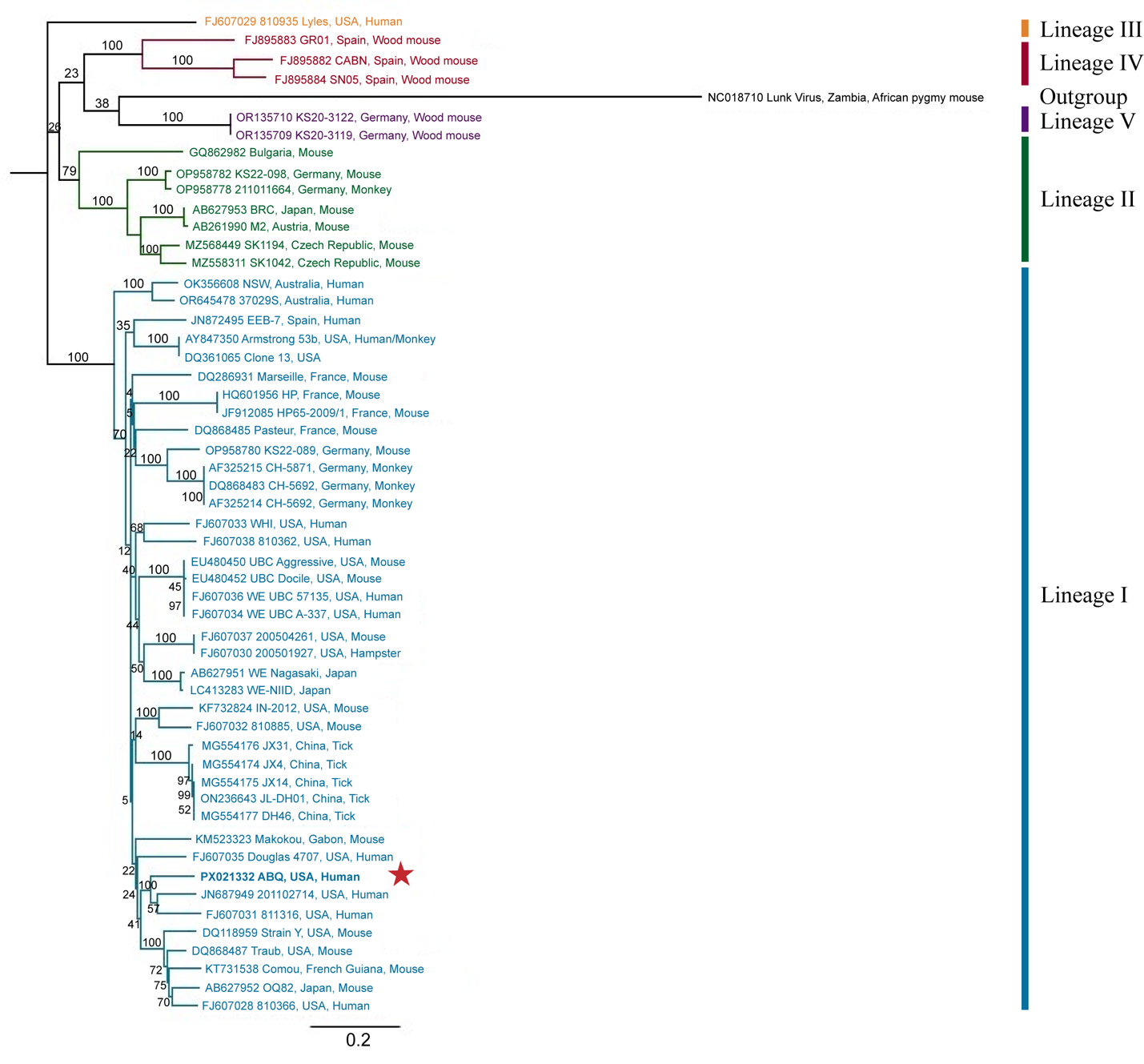


**Supplemental Figure 4:** Phylogenetic analysis of the full-length S segment of LCMV sequences using Maximum Likelihood. This study's LCMV-ABQ S segment sequence was submitted to GenBank (accession # PX021332). LCMV-ABQ is bolded and designated with a red star. Bootstrap values were used to assess node support. Lunk virus from *Mus minutoides* was used as an outgroup. Names of LCMV strains are composed of GenBank accession number, strain name, country of origin/isolation, and host species, if known. The tree was generated using the RAxML plugin in Geneious Prime 1.2 using a GTR model with a gamma distribution and invariable sites and ran for 1000 bootstrap replicates.


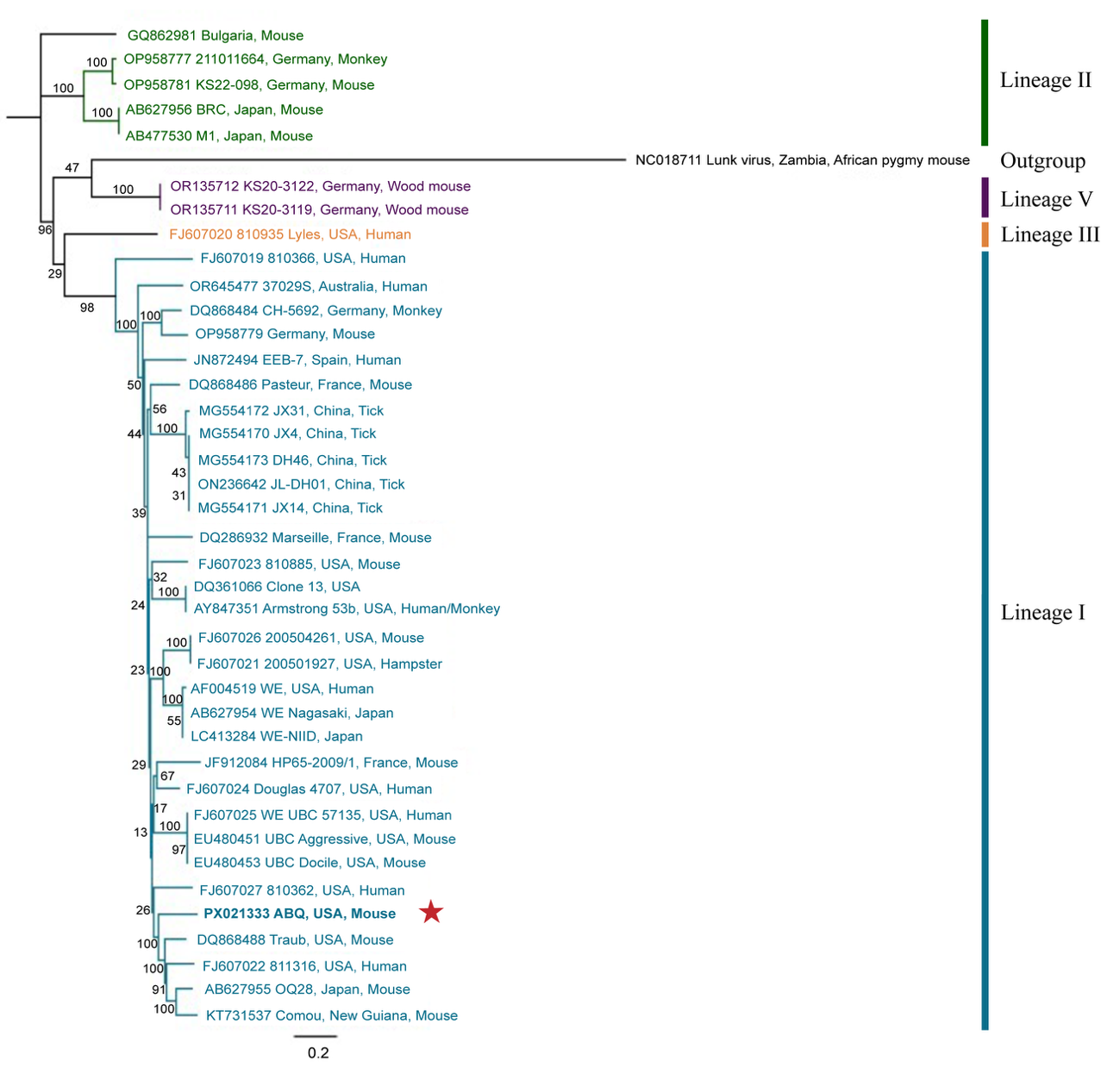


**Supplemental Figure 5:** Phylogenetic analysis of the full-length L segment of LCMV sequences using Maximum Likelihood. This study's LCMV-ABQ L segment sequence was submitted to GenBank (accession # PX021333). LCMV-ABQ is bolded and designated with a red star. Bootstrap values were used to assess node support. Lunk virus from *Mus minutoides* was used as an outgroup. Names of LCMV strains are composed of GenBank accession number, strain name, country of origin/isolation, and host species, if known. The tree was generated using the RAxML plugin in Geneious Prime 1.2 using a GTR model with a gamma distribution and invariable sites and ran for 1000 bootstrap replicates.

**Bayesian phylogenetic analysis**

Convergence of the MCMC chains was assessed using PSRF values and trace plots. The maximum PSRF value across all model parameters was less than the threshold of 1.1, indicating satisfactory convergence. Trace plots showed good mixing. Summary parameter values are included in the supplemental excel file.
